# Development of an Angular Stiffness Sensor to Measure Dental Implant Stability In Vitro

**DOI:** 10.3390/s24216959

**Published:** 2024-10-30

**Authors:** Weiwei Xu, Yen-Wei Chen, Kanako Nagatomo, Yifeng Liu, Jihai Zhou, Michel Dard, I. Y. Shen

**Affiliations:** 1 Department of Mechanical Engineering, University of Washington, Seattle, WA 98195-2600, USA; ishen@uw.edu; 2 Department of Restorative Dentistry, University of Washington, Seattle, WA 98195-7456, USA; ywchen@uw.edu; 3 Nagatomo Periodontics, Shoreline, WA 98133-4034, USA; 4 Quiver Dental Inc., Seattle, WA 98105-5125, USA; yliu@quiver-dental.com (Y.L.);; 5 Department of Electrical & Computer Engineering, University of Washington, Seattle, WA 98195-2500, USA; jihaizho@andrew.cmu.edu

**Keywords:** dental implants, stability, flex constants, angular stiffness, finite element analysis

## Abstract

This investigation aims to develop an angular stiffness sensor intended for measuring dental implant stability in bone. The sensor hardware included a tiny eccentric motor and an accelerometer to measure a flex constant of an implant with its abutment. The sensor software included a mechanics-based model to convert the flex constant to angular stiffness at the implant/abutment junction to indicate the stability. The sensor’s accuracy and effectiveness are demonstrated through use of Sawbones slab models that mimic a mandibular premolar section. The models include a Branemark Mk III implant inserted into Sawbones slabs of 5 different densities with a locator abutment. An incremental insertion torque was first recorded while the implant was placed in the Sawbones models. Then benchtop experiments were conducted to measure resonance frequencies and angular stiffness. Results indicated that angular stiffness increased with Sawbones density, showing high correlation with the measured resonance frequency (R=0.977) and the incremental insertion torque (R=0.959). Finally, accuracy of the angular stiffness sensor is calibrated in light of the resonance frequency. Angular stiffness scores 99% and 95% accuracy for Sawbones models mimicking medium cancellous bones with and without a cortical layer, respectively.

## 1. Introduction

Dental implant stability refers to the immobility of a dental implant in its surrounding alveolar bone [1,2]. A strong functional and structural connection between the implant and the bone results in a high degree of immobility. Therefore, stability has become a notion to indicate successful osseointegration [1,3]. High implant stability at different stages of an implant treatment is a vital sign indicating well-being of the implant [1,4,5].

There are strong needs for objective and reliable implant stability measurements because of their potential benefits. Once implant stability becomes a scientific and accurate measurement, it will help dental professionals make a better assessment of an implant’s health, minimize the risk of complications and failures, assure quality and smooth transition to prosthodontists for crown placement, and identify appropriate timing to crown high-risk patients (e.g., ones with diabetes or osteoporosis). For patients, accurate stability measurements can reduce their anxiety and fears toward implant failures, provide peace of mind, and enhance their experience. Despite the needs, accurate and objective implant stability measurements are yet to be found.

Currently, there are two schools of thought behind stability measurements: impact duration and resonance frequency analysis (RFA). Periotest is a representative product that measures impact duration to infer stability. Periotest operates by mechanically tapping a small mass on an abutment or a crown. Then it measures the contact duration of the tap between the mass and the abutment/crown. Periotest further hypothesizes that the contact duration indicates implant stability. In view of known mechanics principles, contact duration strongly depends on the restitution coefficients of the two tapping surfaces. Contact duration is remotely related to implant stability, which is the bonding strength between the implant and its surrounding bone [6]. Measurements from Periotest are often very scattered, rending accurate measurements extremely difficult [6].

RFA is a well-known method to measure implant stability [1,3,5,7]. The mechanics behind RFA are straightforward. The structural and functional connection resulting from osseointegration changes stiffness at the bone–implant interface, thus affecting resonance frequencies of the bone–implant system. By monitoring a resonance frequency, one can deduce stability changes hoping to indicate the progress of osseointegration. Osstell and Penguin are two RFA-based products available on the market for clinical use [8]. After the resonance frequency is measured, it is converted to an implant stability quotient (ISQ) to indicate stability through an undisclosed algorithm. The effectiveness and accuracy of ISQ measurements are mixed in the literature [9,10]. Some researchers report that ISQ successfully indicates implant stability [5,11,12,13] but others do not [14,15,16,17,18,19,20]. Clinically, RFA is more successful in monitoring the stability change, instead of the stability itself, for a singular implant on a single patient over time [21,22].

Recently, angular stiffness (unit: N·m) at the neck of an implant in bone has demonstrated a high correlation to implant stability in benchtop lab tests [23,24,25]. Conceptually, angular stiffness is the ability of the bone to hold the implant against bending at the implant/abutment junction [23,24,25]. Higher bone–implant bonding strength implies higher ability to withstand bending [1], thus resulting in higher angular stiffness. Since angular stiffness is a real physical quantity, not an artificial index, it can be measured using a standardized specimen via a standardized operating procedure. Therefore, angular stiffness measurements can be repeated and verified by anyone and anywhere. That means angular stiffness measurements can be calibrated, traced, and verified scientifically, independently, and objectively.

Despite these promising results, a rigorous angular stiffness sensor is not yet available to measure stability of dental implants. Although an angular stiffness sensor was shown feasible in [25], there was no scientific procedure to calibrate such a sensor to ensure its accuracy. Moreover, effectiveness of the angular stiffness sensor must be (but not yet) proven in a manner acceptable to dental professionals. Motivated by these needs, this investigation aims to develop one such sensor with the needed accuracy and effectiveness by addressing the following challenges. First of all, this paper discloses a rigorous and systematic way to calibrate the angular stiffness sensor by using a resonance frequency that is independently measured to establish accuracy. Next, this paper demonstrates the effectiveness of the angular stiffness sensor by using generally accepted principles in implant dentistry, such as higher bone density or a higher insertion torque that implies higher stability [26,27]. Experimental results in this paper indicate that higher bone density and higher insertion torque do lead to higher angular stiffness measured, thus proving the effectiveness of the angular stiffness sensor to indicate implant stability.

## 2. Materials and Methods

Direct measurements of angular stiffness are challenging for several reasons. First, the definition of angular stiffness requires a moment divided by an angle, both of which are difficult to measure directly. Second, angular stiffness is a static property, whereas a static load and a small static displacement are difficult to apply and measure in the limited space of an oral cavity with a lot of rigid-body motion (e.g., movement of mandible). Therefore, an indirect measurement is used in this study to obtain angular stiffness instead. First, a low-frequency sinusoidal force is applied, and a corresponding sinusoidal acceleration is measured to form a displacement-to-force ratio (aka “flex constant”). The flex constant is then converted to angular stiffness using a mechanics-based model. When the frequency is low enough (sufficiently below the resonance frequency), the angular stiffness measured via this “dynamic” setting will resemble the measurements in a “static” environment [25]. Implementation of the indirect angular stiffness measurements is explained in detail as follows.

### 2.1. Test Models

**Figure 1:**
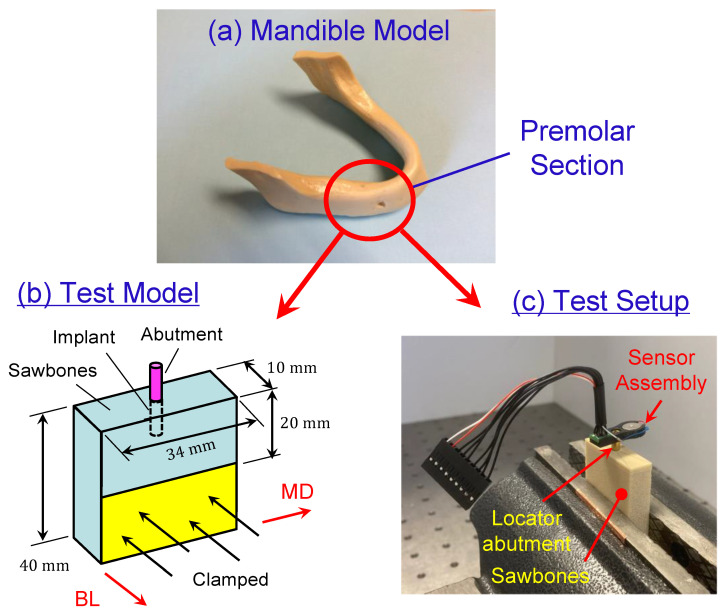
Sawbones test models. (**a**) The test models are designed to mimic a premolar section of a mandible model. (**b**) The test model has dimensions 34×10×40 mm^3^. The longitudinal side with 34 mm mimics the mesial-distal direction, while the thickness side with 10 mm mimics the buccal-lingual direction. The model is clamped such that only 20 mm height is above the clamp. (**c**) In angular stiffness tests, the test model is clamped in a vise and an angular stiffness sensor set is press-fit onto a locator abutment.

The test models consisted of five synthetic bone blocks (Sawbones (Vashon Island, WA, USA)) with densities of 40-PCF (0.64 g/cm^3^), 30-PCF (0.48 g/cm^3^), 20-PCF (0.32 g/cm^3^), 15-PCF (0.24 g/cm^3^), and 40/20-PCF, respectively. All the test models, except the 40/20-PCF model, had dimensions 34 × 10 × 40 mm^3^; see Figure 1b. The 40/20-PCF model had dimensions 34 × 10 × 42 mm^3^ because it had a 2 mm 40-PCF laminate on top of a 40 mm 20-PCF block. The dimensions of the Sawbones were chosen to mimic a premolar section of a mandible (Figure 1a). The longitudinal side with 34 mm mimicked the mesial-distal direction, while the thickness side with 10 mm mimicked the buccal-lingual direction.

Branemark Mk III implants (Nobel Biocare, Switzerland), regular platform, 4 mm in diameter and 11.5 mm in length, were placed at the center of the 34 × 10 mm^2^ surface for each test model; see Figure 1b. The following implantation protocol was adopted in placing all the implants.

Step 1. Used a ∅ 2.3 mm round bur at 800 rpm to create a small spherical cavity at the center of the 34 × 10 mm^2^ surface to mark the implantation site.

Step 2. Used a ∅ 3.2 mm pilot drill at 500 rpm to create a preparation depth of 11.5 mm.

Step 3. Placed the implant by applying a medium pressure. Started the implant placement with a torque of 5 N·cm. If the torque was not enough to advance the implant, increase the torque by an increment of 5 N·cm until the threaded portion of the implant went into the Sawbones test model completely. Recorded the final torque needed. The final torque recorded is defined as the “incremental insertion torque” for the rest of the paper. It is the minimal insertion torque needed for an implant to seat in a test model.

After the implant was placed, a locator abutment with a 4.0 mm platform diameter and 6.0 mm cuff height (Zest, Burbank, CA, USA) was tightly attached onto the implant by a hand torque. Then the test models were clamped partially in vise (unclamped volume 10×20×34 mm^3^) for subsequent tests; see Figure 1b for schematics and Figure 1c for angular stiffness measurements.

### 2.2. Resonance Frequency Measurements

As a reference, resonance frequencies of each Sawbones test model (i.e., Sawbones–implant–abutment assembly) were first measured using the following 3-step protocol [28]. This is a well-established procedure in modal testing. First, an impact hammer applied an impulsive force laterally (7–10 N) to the locator abutment. Next, a laser Doppler vibrometer (LDV) measured the velocity of the locator abutment. Finally, the force and velocity were processed by a spectrum analyzer to obtain a frequency response function (FRF). The frequency at which a sharp peak appeared in the FRF was recorded as a resonance frequency. Since the Sawbones test models presented multiple resonance frequencies, the lowest one was defined as “the resonance frequency” for the rest of the paper. This vibration mode corresponds to the bending of the test model in the buccal-lingual direction (along the 10 mm side). Since this resonance frequency is measured entirely from experiments, it is denoted as $\omega_{\mathrm{Exp}}$. It also serves as an independent reference for calibration of the angular stiffness sensor.

### 2.3. Angular Stiffness Sensor via Accelerometer

The angular stiffness sensor is a model-based measurement tool supported by rigorous mechanics principles. Angular stiffness sensing is a two-step process: measurement (via hardware) and extraction (via software). In the hardware measurement step, a known force is first applied to an implant via its abutment. Then the motion of the abutment is measured to obtain a displacement–force ratio, also known as a “flex constant” [29]. In the software extraction step, a finite element model is created to process the measured flex constant so as to extract angular stiffness of the bone–implant–abutment system. Setups of the hardware measurement and software extraction for the five Sawbones test models are explained in detail as follows.

**Figure 2:**
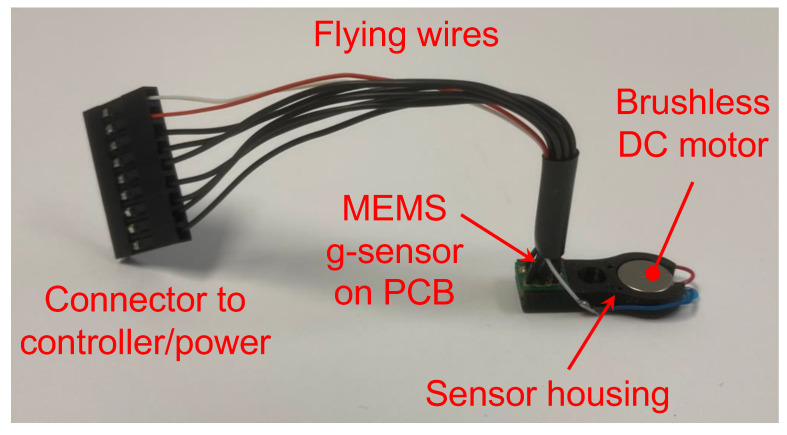
The sensor assembly built to measure the flex constants comprised a brushless DC motor, a MEMS-based accelerometer (aka *g*-sensor), a sensor housing, a set of flying wires, and a connector to a controller and a power source. The motor and the accelerometer were seated snugly in the housing. Through a central hole of the housing, the angular stiffness sensor set was press-fit onto the locator abutment. The motor generated a known eccentric force, gently wiggling the locator abutment. The accelerometer measured the acceleration of the locator abutment. The measured acceleration was forwarded to the controller for post-processing to extract angular stiffness.

Step 1. Measuring Flex Constants. Figure 2 shows the angular stiffness sensor assembly that was built to measure the flex constants. The sensor assembly included a motor, an accelerometer (aka g-sensor), a sensor housing, a set of flying wires, and a connector to a controller with a power source. The sensor housing was made of plastics and was 3-D printed. The motor and the accelerometer were assembled and snugly seated in the sensor housing. The housing also had a central hole, through which the sensor assembly was pressed-fit onto the locator abutment; see Figure 1c. Also, the axial location of the sensor assembly was measured and recorded for later use; see Table 1. The axial location is defined as the axial distance from the top surface of the Sawbones model to the center of the motor and accelerometer, respectively.

The motor was a brushless DC coin vibrator motor (Vybronics, VW0825AB001G, ∅ 8 mm × 2.5 mm). The motor generated an eccentric force (typically in the frequency range of 250–350 Hz depending on the voltage and test models used) given by

(1) $$F=mr\omega^{2}$$

where m was the eccentric mass of the motor, r was the eccentricity of the mass, and ω was the motor speed. In particular, m and r were measured from the motor, and ω was measured through the acceleration. Therefore, the exact force acting on the locator abutment was identified. The unbalance of the motor used was $mr=8.5\times 10^{-8}$ kg·m. The unbalance was reverse-engineered and also calibrated using a proof mass.

The accelerometer was a MEMS-based accelerometer (Bosch Sensortec, Digital triaxial acceleration sensor BMA456). It was surface-mounted onto a rigid printed circuit board (PCB), with an overall footprint roughly 3.0 mm × 3.0 mm. As the eccentric motor rotated, the measured acceleration was sinusoidal with an amplitude a and a frequency ω. Therefore, the displacement δ at the locator abutment was found as

(2) δ=aω2

**Figure 3:**
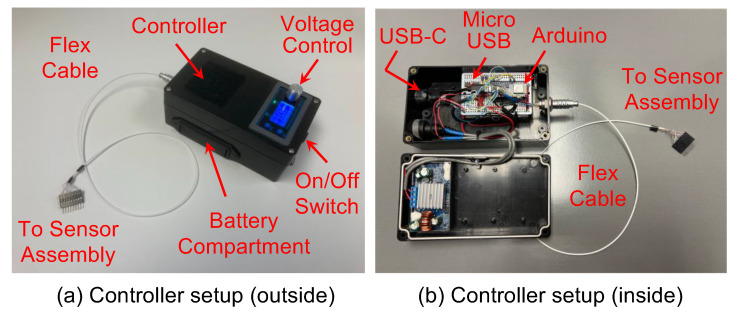
The controller setup. (**a**) The controller was battery-powered and had a voltage adjustment knob to control the motor speed. A flex cable connected the controller and the sensor assembly to power the motor and accelerometer. (**b**) Inside the controller, an Arduino micro-processor was programmed to read the measured acceleration digitally via the flex cable. The Arduino micro-processor then transferred the measured acceleration via Bluetooth or a USB for subsequent data processing.

Figure 3a shows the controller that powered the motor and the accelerometer. The controller was battery-powered and had a voltage adjustment knob to control the motor speed. A flex cable connected the controller and the sensor assembly to power the motor and accelerometer. Inside the controller (Figure 3b), an Arduino micro-processor (Arduino Nano 33 BLE) was programmed to read the measured acceleration digitally via the flex cable. The Arduino micro-processor then transferred the measured acceleration to a mobile app via Bluetooth or to a computer via a USB for data processing.

The operating procedure to measure the flex constants was straightforward. First, the motor generated a known eccentric force F (cf. Equation (1)), gently wiggling the locator abutment whose acceleration a was measured by the accelerometer. For the data processing afterwards, a nonlinear regression algorithm [25] was used to extract the frequency ω from the measured acceleration a. According to Equations (1) and (2), an experimentally measured displacement–force ratio δFExp was calculated via

(3) δFExp=amrω4

Since the motor spins at a frequency much lower than the resonance frequency of the test model, the measured displacement–force ratio is almost the same as the flex constant found in [29] under a static load. For the rest of the paper, “displacement–force ratio” and “flex constant” will be used interchangeably to denote δF, which could be measured experimentally or predicted via a finite element model in Step 2 below.

Step 2. Extracting Angular Stiffness. A finite element model simulating the test models is created to convert the measured flex constant δFExp to angular stiffness. This is the software extraction step of the angular stiffness sensing. The detailed process is explained as follows.

**Figure 4:**
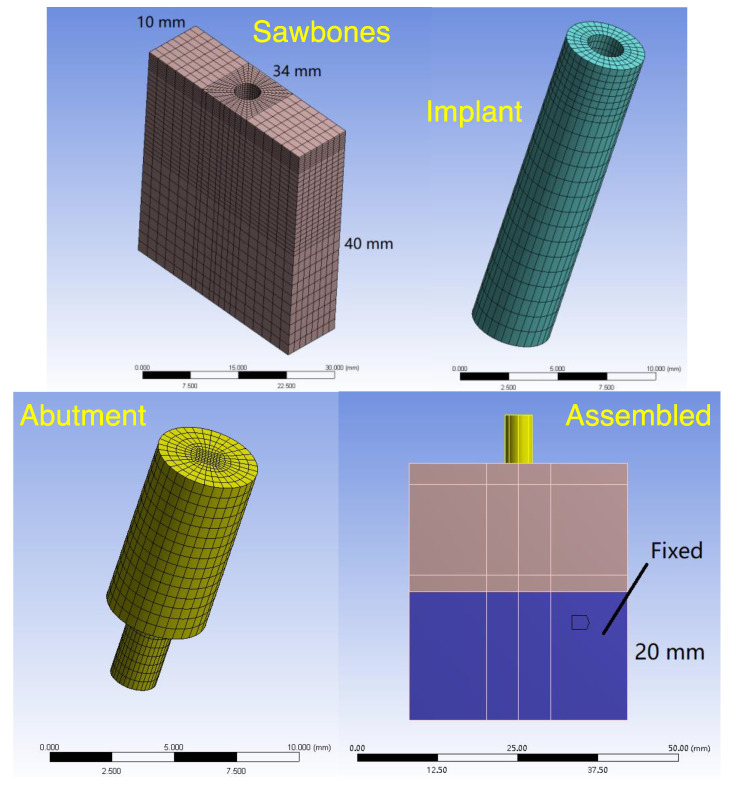
The finite element model comprised a Sawbones portion, an implant portion, and a locator abutment portion. The three portions were assembled together and the lower 20 mm region was fixed to form the finite element model. The experimentally measured displacement–force ratio δFExp at the abutment was then fed into the finite element model to estimate local stiffness of the Sawbones, which, in turn, generated the angular stiffness of the bone–implant–abutment system at the implant–abutment junction.

Step 2.1: The Finite Element Model. Figure 4 shows the structure of the finite element model. The finite element model comprised a Sawbones portion, an implant portion, and a locator abutment portion, reflecting the test model, implant, and locator abutment used in the experiments, respectively. To form the complete finite element model, the three portions were assembled together. Moreover, the lower 20 mm region was fixed to reflect the clamp from the vise (cf. Figure 1c).

**Figure 5:**
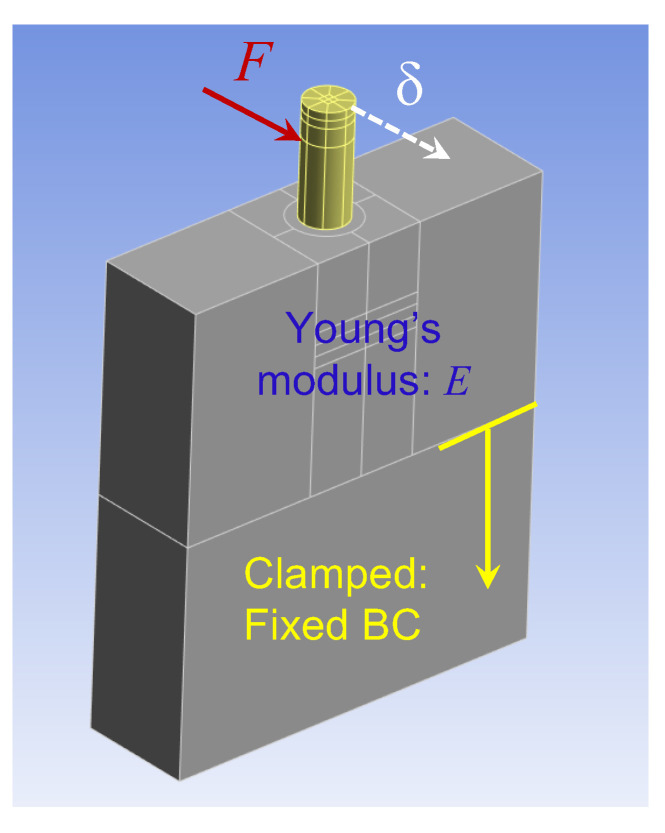
Figure 5 shows how the finite element model was used to generate the flex constant via a static analysis. First, a force F was applied to the locator abutment at the location of the motor, whereas the displacement δ was calculated at the location of the accelerometer. Then the ratio of the displacement δ to the force F gave the calculated flex constant δFFEA, which depended on Young’s modulus E of the slab.

Figure 5 shows how the finite element model was used to generate a theoretical flex constant δFFEA via a static analysis. First, a force F was applied to the locator abutment at the location of the motor, whereas the displacement δ was calculated at the location of the accelerometer (cf. Table 1). The ratio of the displacement δ to the force F was the flex constant δFFEA, which depended on Young’s modulus E of the Sawbones portion. Further, the Young’s modulus E was determined by adjusting the value of E such that the calculated flex constant δFFEA matched the measured flex constant δFExp. In this regard, the Young’s modulus E obtained was an equivalent modulus that incorporated (and homogenized) all stiffness effects, such as Sawbones’ elasticity, clamping interfaces at the vise, interference between the implant and the Sawbones slab, and internal stresses of the Sawbones slab [25].

**Figure 6:**
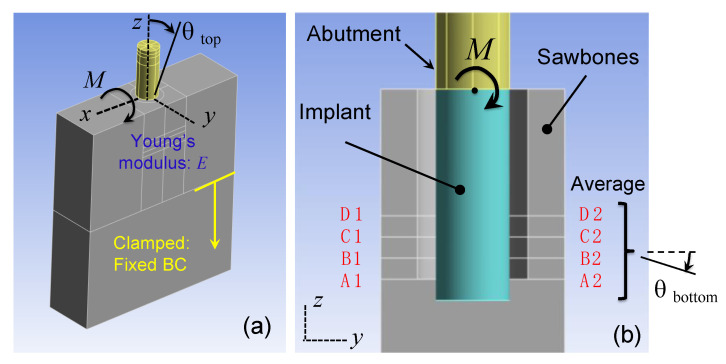
Determining angular stiffness $k_{\theta }$. (**a**) A moment M was applied at the implant–abutment junction. The implant and the Sawbones slab deformed and rotated accordingly. Let $\theta_{\mathrm{top}}$ be the angle of rotation at the top of the implant (i.e., implant–abutment junction). (**b**) Let $\theta_{\mathrm{bottom}}$ be the angle of rotation at the bottom region of the implant. The angular stiffness was defined as $k_{\theta }=M/(\theta_{\mathrm{top}}-\theta_{\mathrm{bottom}})$. To better represent $\theta_{\mathrm{bottom}}$, four planes at the bottom region were chosen. Their averaged angle of rotation produced $k_{\theta }$.

**Figure 7:**
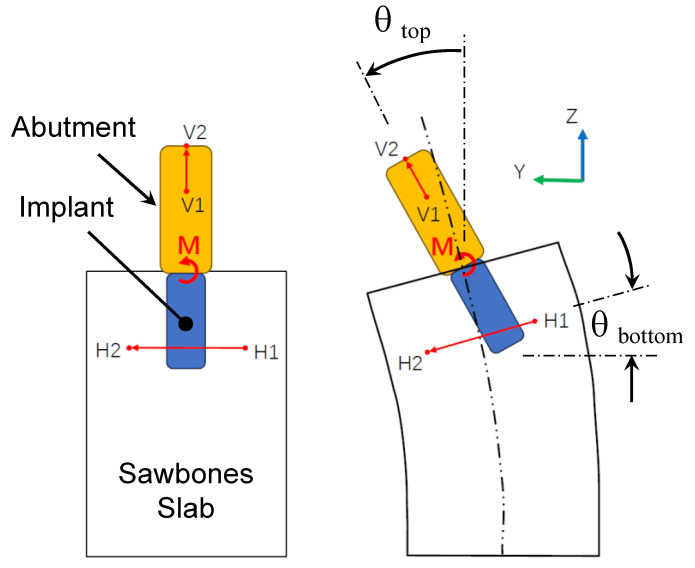
The moment M causes the implant and the Sawbones slab to deform and rotate. Every point in the implant and the surrounding slab rotates differently. $\theta_{\mathrm{top}}$ and $\theta_{\mathrm{bottom}}$ are the angle of rotation at the top of the implant (i.e., implant–abutment junction) and the bottom region of the implant. V1–V2 and H1–H2 are segments of the implant centerline and Sawbones slab. The rotation of segment V1–V2 defines $\theta_{\mathrm{top}}$, while the rotation of segment H1–H2 defines $\theta_{\mathrm{bottom}}$.

Step 2.2: Conversion to Angular Stiffness. With the Young’s modulus E found in Step 2.1, a moment M was applied instead at the implant–abutment junction as shown in Figure 6a to calculate the corresponding angular stiffness. As a result of the moment M, the implant and the Sawbones slab deformed and rotated; see Figure 7. Every point in the implant and the surrounding slab, however, rotated differently. As shown in Figure 7, let $\theta_{\mathrm{top}}$ be the angle of rotation at the top of the implant (i.e., implant–abutment junction) where the moment M was applied. To determine $\theta_{\mathrm{top}}$, let V1–V2 be a segment of the implant center. The rotation of segment V1–V2 was $\theta_{\mathrm{top}}$. Similarly, let $\theta_{\mathrm{bottom}}$ be the angle of rotation at the bottom region of the implant. Also, let H1–H2 be a segment of the Sawbones slab in the bottom region of implant. The rotation of segment H1–H2 defines $\theta_{\mathrm{bottom}}$.

Then, an implant angular displacement was defined as

(4) $$\theta =\theta_{\mathrm{top}}-\theta_{\mathrm{bottom}}=\frac{\pi }{2}-\cos^{-1}\left(\frac{\vec{V_{1}V_{2}}\cdot \vec{H_{1}H_{2}}}{\left|V_{1}V_{2}\right|\left|H_{1}H_{2}\right|}\right)$$

After the deformed coordinates of segments V1–V2 and H1–H2 were obtained from the finite element analysis of Figure 6a, Equation (4) was used to determine angular displacement θ. Lastly, the angular stiffness $k_{\theta }$ at the implant–abutment junction was defined as

(5) $$k_{\theta }\equiv \frac{M}{\theta }=\frac{M}{\theta_{\mathrm{top}}-\theta_{\mathrm{bottom}}}$$

In the bottom region of the implant, the angle of rotation of segment H1–H2 varied considerably point by point thus making angular stiffness $k_{\theta }$ unnecessarily sensitive via Equation (5). To better represent $\theta_{\mathrm{bottom}}$, an averaged angle of rotation from four sections was used; see Figure 6b. The four sections A1-A2 to D1-D2 corresponded to 90%, 80%, 70%, and 60% of the implant length. The angle of rotations of these four planes were averaged to obtain $\theta_{\mathrm{bottom}}$.

### 2.4. Confirming Angular Stiffness Measurements via LDV

To confirm the accuracy of the acceleration measurements, an LDV was also used to measure the velocity v of the abutment as a reference in conjunction with the sensor assembly. Specifically, a laser beam from the LDV was projected laterally onto the locator abutment between the sensor assembly and the Sawbones model as shown in Figure 1c. The laser beam was oriented such that it was parallel to the sensor assembly to give consistent measurements thereof. The axial location of the LDV measurements (i.e., axial distance from the laser spot and the top surface of the Sawbones model) was also shown in Table 1. Once the motor frequency ω was found, the velocity v was converted to displacement δ via

(6) δ=vω

The displacement δ from the LDV was then used to obtain an experimentally measured flex constant δFExp, from which a Young’s modulus E was extracted. Based on the extracted Young’s modulus E, an angular stiffness $k_{\theta }$ was obtained using the same Step 2.1 and Step 2.2 above.

### 2.5. Sensitivity Study

**Figure 8:**
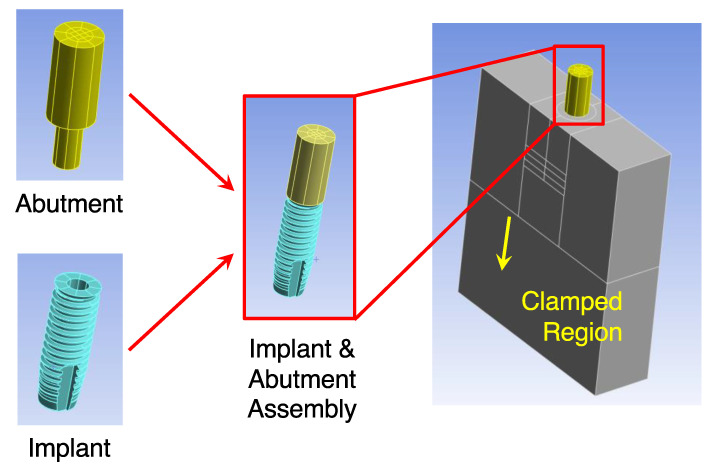
Effect of the finite element model on angular stiffness $k_{\theta }$. A second finite element model was created with detailed modeling of the threads. The rest was identical to the first finite element model. The second model then converted the experimentally measured flex constant δFExp into angular stiffness $k_{\theta }$ using the procedures explained in Steps 2.1 and 2.2.

To study how sensitive the finite element model would affect the extracted angular stiffness, a second finite element model was introduced; see Figure 8. It included detailed modeling of the threads, whereas the first one did not (cf. Figure 4). In particular, the outside diameter of the threads was 4 mm (same as the cylinder diameter). As a result, the threaded implant had a smaller volume than the cylindrical implant. To investigate the effects of the volume reduction, two versions of the threaded model were considered. In the first version, the threaded implant had the same mass as the cylindrical implant in Figure 4. In the second version, the threaded implant had the same density as the cylindrical implant in Figure 4. In the first version, the transition from the cylindrical implant to the threaded implant only affected the stiffness of the implant. In the second version, the transition affected both the stiffness and inertia of the implant. Both versions of the second finite element model were then used to convert the experimentally measured flex constant δFExp into angular stiffness $k_{\theta }$ using the procedures explained in Steps 2.1 and 2.2.

## 3. Results

### 3.1. Angular Stiffness Measurements via Accelerometer

**Figure 9:**
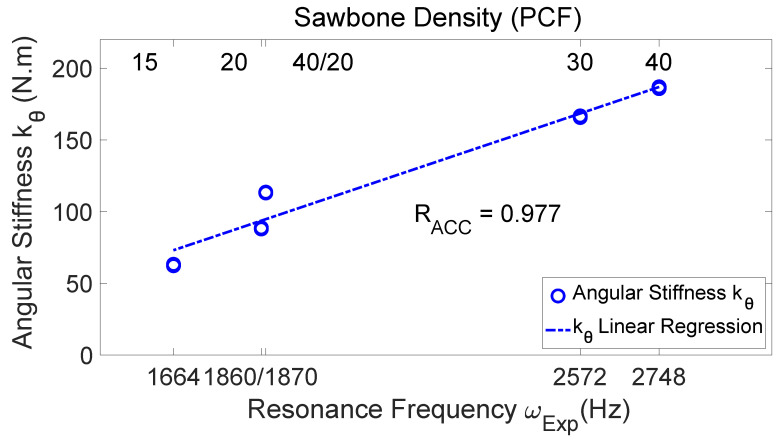
The measured angular stiffness of the test models versus the resonance frequency. The bottom abscissa is the resonance frequency of the test models, which increases as the bone density increases (as shown in the top abscissa). The measured angular stiffness values have high correlation with the resonance frequency (R=0.977).

Figure 9 presents the measured angular stiffness of the five test models versus the measured resonance frequency. The bottom abscissa is the resonance frequency $\omega_{\mathrm{Exp}}$ of the test models (i.e., the resonance frequency measured by the hammer and LDV without the angular stiffness sensor seated on the locator abutment as a reference). Also labeled in Figure 9 is the Sawbones density associated with each resonance frequency (see the top abscissa). According to Figure 9, the measured angular stiffness values increase as the resonance frequency (and also the bone density) increase, with a high linear correlation coefficient ($R_{\mathrm{ACC}}=0.977$).

**Figure 10:**
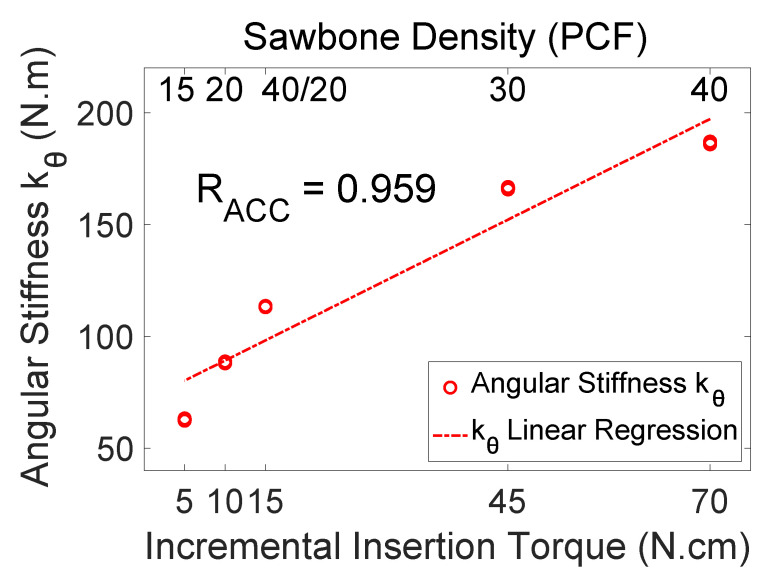
The measured angular stiffness of the test models versus the incremental insertion torque. The abscissa is the incremental insertion torque of the test model, which increases as the bone density increases. The measured angular stiffness values have high correlation with the incremental insertion torque (R=0.959).

Figure 10 shows the angular stiffness (converted from the measured flex constant) of the test models versus the incremental insertion torque measured from each test model. The indirectly measured angular stiffness values have high correlation with the incremental insertion torque ($R_{\mathrm{ACC}}=0.959$). Note that the incremental insertion torque measurements had a coarse resolution and therefore less accuracy than the resonance frequency measurements. It is natural that the correlation coefficient is slightly smaller for the incremental insertion torque.

Table 2 compares the measured angular stiffness (in N·m), displacement–force ratio (in μm/N), and resonance frequencies (in Hz) for various Sawbones models under the same clamping force (These are the data supporting Figure 9 and Figure 10). The incremental insertion torque (in N·cm) is also listed for reference. The angular stiffness and displacement–force ratio appear as an average plus and minus a half-range. The small range values indicate that the data are not scattered.

### 3.2. Angular Stiffness Measurements via LDV

**Figure 11:**
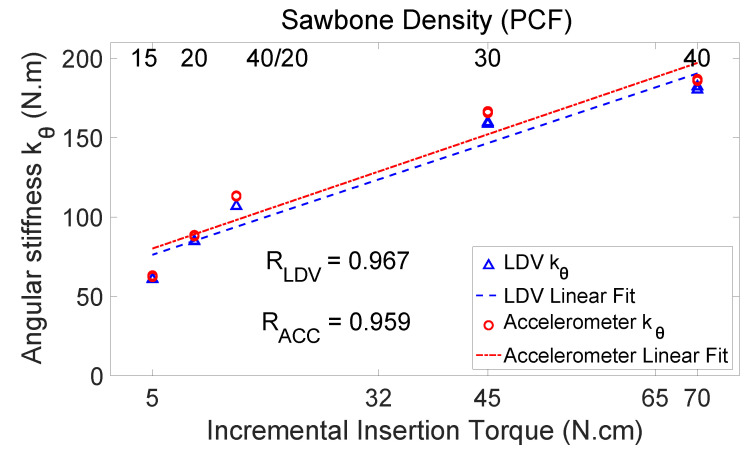
The angular stiffness measured from the accelerometer and the LDV. The abscissa is the insertion torque. The angular stiffness values measured from the LDV have slightly higher correlation with the incremental insertion torque ($R_{\mathrm{LDV}}=0.967$). The high R values from the accelerometer and the LDV prove the accuracy of the accelerometer.

Figure 11 compares angular stiffness values measured from the accelerometer and from the LDV. The abscissa is the insertion torque. The two measured angular stiffness values are very close. The angular stiffness values measured from the LDV also have high correlation with the incremental insertion torque (R=0.967). The high R values and close agreement of the accelerometer and the LDV measurements prove the accuracy of the accelerometer in measuring angular stiffness. The close agreement of the LDV and accelerometer measurements also imply that PCB’s inertia and stiffness have negligible effects on the measurement accuracy.

### 3.3. Sensitivity Study Results

Table 3 compares angular stiffness $k_{\theta }$ extracted via the two finite element models (cf. Figure 4 and Figure 8) using the same set of measured flex constants δFExp. Table 3 compares angular stiffnesses extracted through the cylindrical and threaded implant models. There are several things worth mentioning. First of all, the threaded implant models have a smaller implant volume, thus a smaller angular stiffness (about 20–30% less) than that obtained from the cylindrical implant model. Note that the difference does not mean that one model gives better results than the other. It only means that two models give two different readings, just like a temperature can be read in Celsius or in Fahrenheit. Second, when a threaded model is used, the same-mass version and the same-density version result in the same angular stiffness. This is not surprising, because angular stiffness is a static property determined entirely by the geometry and elasticity and not by the inertia. Lastly, the results indicate that diameter of the implant affects the angular stiffness significantly. Note that the angular stiffness is an overall representation of the elasticity of the implant, the surrounding Sawbones, and the interface thereof. Any reduction in the elasticity of the implant, the surrounding Sawbones, or the interface will decrease the angular stiffness.

## 4. Discussion

The rigor and accuracy of the measured angular stiffness are discussed in depth as follows.

### 4.1. Rigor of Angular Stiffness Measurements

Pagliani et al. have shown that a flex constant has a significant inverse correlation to ISQ [29]. In their study, a lateral force of 25 N was applied on a custom-made abutment at 10 mm above the abutment–implant junction, and the displacement of the abutment in the lateral direction was measured at the same height. Then a flex constant was obtained by calculating the ratio of the measured displacement to the known applied force. When the measured flex constant was 25μm/N, the corresponding ISQ was roughly 55. When the flex constant was about 4 μm/N, the corresponding ISQ was roughly 60–65 [29].

In the current study, the motor and accelerometer were 8.92 mm and 9.62 mm above the abutment–implant junction, respectively, for the 15-PCF test model (cf. Table 1). The corresponding displacement–force ratio was 25.43 ± 0.44 μm/N (cf. Table 2). For the 30-PCF test model, the motor and the accelerometer were 8.60 mm and 9.30 mm above the abutment–implant junction, respectively (cf. Table 1). The corresponding displacement–force ratio was 5.62 ± 0.09 μm/N (cf. Table 2). The displacement–force ratios measured in the current study are totally comparable with the flex constants measured in [29].

There are two major issues of using a flex constant to indicate stability. First, the flex constant changes when the loading point and measuring point are changed. For example, an implant in a Sawbones premolar model (cf. Figure 1c) has a fixed level of stability, but the implant can present different flex constants if the loading point or the measuring point varies. Second, even if the loading point and the measuring point are fixed, the flex constant implies different levels of stability when the model geometry changes, for example, from a Sawbones premolar model (cf. Figure 1c) to a real mandible or a bovine bone. In other words, there is a missing link between the flex constants and the implant stability.

The missing link is the mechanics-based model introduced in the current study to extract the angular stiffness at the implant–abutment junction. There are two unique features here to make this approach rigorous and powerful. First, the mechanics-based model accommodates the changes in the loading point, the measuring point, and the model geometry used. When the ratio δFExp is measured from a Sawbones premolar model (cf. Figure 1c), the mechanics model should replicate the Sawbones premolar model (cf. Figure 4). When the ratio δFExp is measured from a mandible, the mechanics model should replicate the mandible [24]. With the mechanics-based model, one can then convert a flex constant to indicate stability of a dental implant in any use case.

The second unique feature is the use of angular stiffness. With angular stiffness, all flex constants converge to a single physical quantity—through use of the mechanics-based model—to indicate implant stability. This is rather significant, because it has long been an unsolved issue that “implant stability per se has not been defined using any other quantity” [30]. As a result, implant stability is not indicated by a physical quantity that can be calibrated and traced to serve as a reference.

With the mechanics-based model, implant stability is now defined through use of angular stiffness. Since angular stiffness is a physical quantity, it can be measured using a standardized specimen and a standardized operating procedure once a rigorous mechanics model is established. Therefore, angular stiffness measurements can be repeated and verified by anyone and anywhere. That means angular stiffness measurements can be calibrated, traced, and verified scientifically, independently, and objectively across various models and users. It is not an artificial nor an empirical index. Once angular stiffness measurements are calibrated on a standardized model, they can be transferred to any other models (e.g., mandible and maxilla) by using corresponding mechanics models to extract the angular stiffness.

### 4.2. Calibration of Angular Stiffness Sensor

The close agreement between the accelerometer and LDV shown in Figure 11 only proves that the acceleration measurements are accurate. If the finite element models are not accurate, the extracted angular stiffness will not be accurate either. But, what is the accuracy of the finite element models? How can the accuracy of different finite element models be evaluated?

One way to evaluate the accuracy is to use resonance frequencies as follows. In Step 2.1, the Young’s modulus E of the Sawbones model was obtained from the measured flex constants δFExp through the use of a finite element model. This Young’s modulus E together with the finite element model could further generate the lowest resonance frequency $\omega_{\mathrm{FEA}}$. The resonance frequency $\omega_{\mathrm{FEA}}$ was then compared with the experimentally measured resonance frequency $\omega_{\mathrm{Exp}}$. Since $\omega_{\mathrm{FEA}}$ involves the finite element model whereas $\omega_{\mathrm{Exp}}$ is entirely from the experiments, the comparison of these two resonance frequencies represents a measure of the accuracy of the finite element model. If a finite element model was accurate, $\omega_{\mathrm{FEA}}$ and $\omega_{\mathrm{Exp}}$ would agree well with each other.

**Figure 12:**
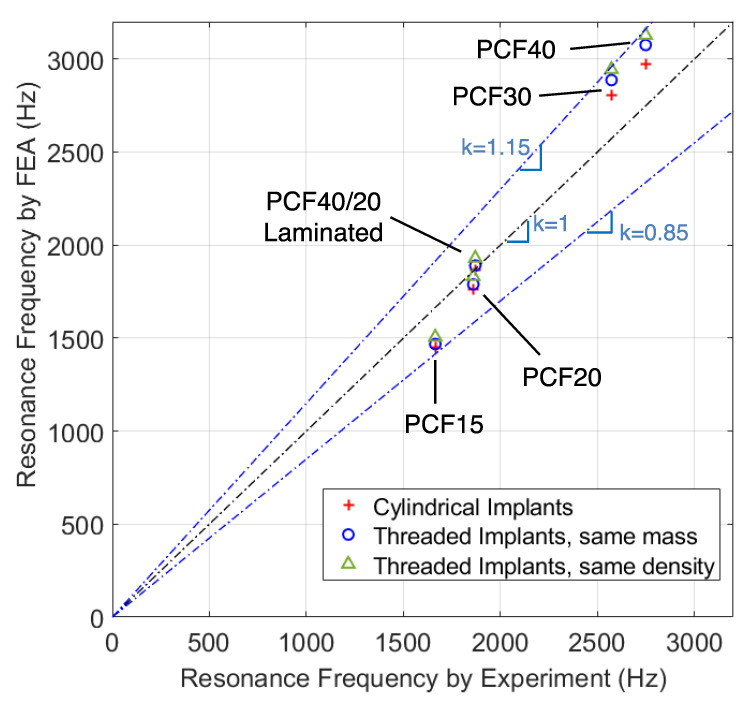
Calibration through use of resonance frequency. When converting the measured flex constants δFExp to angular stiffness using a finite element model, two byproducts are the equivalent Young’s modulus E of the slab and a resonance frequency $\omega_{\mathrm{FEA}}$ derived thereof. Since $\omega_{\mathrm{FEA}}$ involves the use of the finite element model whereas $\omega_{\mathrm{Exp}}$ is purely experimental, comparison of $\omega_{\mathrm{FEA}}$ and $\omega_{\mathrm{Exp}}$ gives a measure of how accurate the finite element is in capturing the complex mechanics behavior inside the Sawbones model.

Table 3 lists the values of $\omega_{\mathrm{FEA}}$ from the finite element models with cylindrical and threaded implants (cf. Table 2 for $\omega_{\mathrm{Exp}}).$ Figure 12 further plots $\omega_{\mathrm{FEA}}$ vs. $\omega_{\mathrm{Exp}}$ based on the values in Table 2 and Table 3. When $\omega_{\mathrm{FEA}}$ is closer to the main diagonal, it means that the model is more accurate because $\omega_{\mathrm{FEA}}$ is closer to $\omega_{\mathrm{Exp}}$. There are several results from Figure 12 worth mentioning. First of all, the accuracy depends on the bone density. For the median bone densities 40/20-PCF and 20-PCF, which were intended to model cancellous bones with and without a layer of cortical bone, $\omega_{\mathrm{FEA}}$ and $\omega_{\mathrm{Exp}}$ agree very well indicating a good accuracy from the finite element models. In contrast, $\omega_{\mathrm{FEA}}$ and $\omega_{\mathrm{Exp}}$ show a 10–15% difference when the bone is extremely soft (PCF-15) or extremely hard (PCF-40).

Another interesting finding is that the cylindrical implant model has higher accuracy than the threaded implant models for PCF40/20, PCF-30, and PCF-40 models. For the threaded models, the same-mass version is slightly more accurate than the same-density version also for PCF40/20, PCF-30, and PCF-40 models. It may seem contra-intuitive why a more detailed and legitimate implant geometry turns out to have a lower accuracy. One possible explanation is that the mechanics inside the tested Sawbones model is far more complicated than the finite element model assumed in this study. In a tested Sawbones model, there is significant interference between the threads and the neighboring Sawbones materials, which can become very nonlinear under the high-strain condition. The clamped boundary condition may also cause significant internal stresses. Sawbones materials are highly nonlinear, with different tensile and compressive Young’s modulus. All these complex mechanics features inside the tested Sawbones model may not be modeled effectively by using an equivalent Young’s modulus E throughout the entire Sawbones slab. After all, the finite element models are, in a way, a sophisticated curve-fitting tool to convert the measured flex constants to angular stiffness by homogenizing the complex internal stress field into an equivalent Young’s modulus E.

As discovered in [23], resonance frequency is not an accurate indicator of dental implant stability. It does not mean resonance frequency measurements in and of itself are totally useless. In fact, resonance frequency is easy to measure, and its measurements are fairly accurate. Therefore, resonance frequency is widely accepted as a reliable physical quantity to validate mechanics models. For example, the authors of [23] used resonance frequency to validate their finite element model. In a much broader context, measuring resonance frequencies is one of the fundamental procedures used in experimental model testing. The fact that resonance frequency does not correlate well to implant stability is irrelevant to using resonance frequency to validate mechanics models.

## 5. Conclusions

In this investigation, an angular stiffness sensor was developed and the following conclusions could be drawn. An experimental measurement of flex constants together with a mechanics-based model can successfully extract a physical quantity of “angular stiffness” to indicate the stability of a dental implant. Measured angular stiffness values have a high correlation to the resonance frequency and the incremental insertion torque of the Sawbones models tested. The angular stiffness sensor can be effectively calibrated through the use of resonance frequencies. Angular stiffness scores 99% and 95% accuracy for Sawbones models mimicking medium cancellous bones with and without a cortical layer, respectively.

## Figures and Tables

**Table 1:** Axial locations of the sensor assembly and LDV are slightly different for each test model. Also, the motor and the accelerometer are not at the same level. The axial location is defined as the axial distance from the top surface of the Sawbones model to the center of motor, accelerometer, and LDV spot, respectively.

Sawbones Density (PCF)	Motor Location (mm)	Accelerometer Location (mm)	LDV Location (mm)
15	8.92	9.62	2.78
20	8.75	9.45	2.87
40/20	8.42	9.12	2.55
30	8.60	9.30	2.85
40	9.02	9.72	3.60

**Table 2:** Comparison of the measured angular stiffness (N·m), displacement–force ratio (μm/N), and resonance frequencies (in Hz) for various Sawbones materials under the same clamping force. The incremental insertion torque (in N·cm) is also listed for reference. The angular stiffness and displacement–force ratio appear in the form of an average plus and minus a half-range (out of 5–10 measurements). The small range values indicate that the data are not scattered.

Sawbones Density (PCF)	Incremental Insertion Torque (N·cm)	Resonance Frequency (Hz)	Angular Stiffness (N·m)	Displacement-Force Ratio (μm/N)
15	5	1664	62.47 ± 0.59	25.43 ± 0.44
20	10	1860	88.36 ± 0.40	15.80 ± 0.05
40/20	15	1870	113.38 ± 0.56	11.93 ± 0.16
30	45	2572	166.08 ± 1.10	5.62 ± 0.09
40	70	2748	186.28 ± 0.60	4.69 ± 0.04

**Table 3:** Comparison of angular stiffness and resonance frequency obtained by two finite element models. The first model simulates the implant as a cylinder, while the second model simulates the implant with threads. Moreover, the threaded model has two versions. One has the same implant mass, but the other has same implant density.

Sawbones Density (PCF)	$\mathrm{Resonance}\;\mathrm{Frequency}\;\omega_{\mathrm{FEA}}$ (Hz)			Angular Stiffness (N·m)		
	Cylinder Model	Thread Model		Cylinder Model	Thread Model	
		Same Mass	Same Density		Same Mass	Same Density
15	1451	1467	1506	62.47	49.34	49.34
20	1762	1789	1831	88.36	68.15	68.15
40/20	1862	1889	1929	113.38	87.09	87.09
30	2807	2886	2944	166.08	123.09	123.09
40	2971	3075	3128	186.28	138.12	138.12

## Data Availability

This study did not report any data.
